# Hyperspectral and Physiological Analyses of Coral-Algal Interactions

**DOI:** 10.1371/journal.pone.0008043

**Published:** 2009-11-26

**Authors:** Katie Barott, Jennifer Smith, Elizabeth Dinsdale, Mark Hatay, Stuart Sandin, Forest Rohwer

**Affiliations:** 1 Department of Biology, San Diego State University, San Diego, California, United States of America; 2 Scripps Institution of Oceanography, University of California San Diego, La Jolla, California, United States of America; University of Hyderabad, India

## Abstract

Space limitation leads to competition between benthic, sessile organisms on coral reefs. As a primary example, reef-building corals are in direct contact with each other and many different species and functional groups of algae. Here we characterize interactions between three coral genera and three algal functional groups using a combination of hyperspectral imaging and oxygen microprofiling. We also performed *in situ* interaction transects to quantify the relative occurrence of these interaction on coral reefs. These studies were conducted in the Southern Line Islands, home to some of the most remote and near-pristine reefs in the world. Our goal was to determine if different types of coral-coral and coral-algal interactions were characterized by unique fine-scale physiological signatures. This is the first report using hyperspectral imaging for characterization of marine benthic organisms at the micron scale and proved to be a valuable tool for discriminating among different photosynthetic organisms. Consistent patterns emerged in physiology across different types of competitive interactions. In cases where corals were in direct contact with turf or macroalgae, there was a zone of hypoxia and altered pigmentation on the coral. In contrast, interaction zones between corals and crustose coralline algae (CCA) were not hypoxic and the coral tissue was consistent across the colony. Our results suggest that at least two main characteristic coral interaction phenotypes exist: 1) hypoxia and coral tissue disruption, seen with interactions between corals and fleshy turf and/or some species of macroalgae, and 2) no hypoxia or tissue disruption, seen with interactions between corals and some species of CCA. Hyperspectral imaging in combination with oxygen profiling provided useful information on competitive interactions between benthic reef organisms, and demonstrated that some turf and fleshy macroalgae can be a constant source of stress for corals, while CCA are not.

## Introduction

Coral reef ecosystems are among the most diverse and threatened ecosystems on the planet. Estimates suggest that 20% of the world's coral reefs have already been lost, with another 50% likely to be lost in the near future [1] due to a variety of human influences [1]–[3]. Local factors such as overfishing, habitat destruction and pollution from terrestrial runoff (e.g. eutrophication) are causing direct destruction of reefs. Global threats such as rising sea surface temperatures have led to widespread bleaching events [4] and coral disease has emerged as a critical problem [5], [6]. Other climate related stressors such as ocean acidification may lead to loss of reef structure [2], [7]. While the impacts of local threats may be reduced through management action, global threats to coral reefs are likely to increase in severity in the coming years [2], [8].

Disturbed coral reefs are typically characterized by loss of coral cover followed by an increase in the abundance of fleshy algae (turf and macroalgae), a phenomenon that has been termed the coral-algal phase shift [9], [10]. There are a wide variety of factors that can work in concert to lead to a coral-algal phase shift. In the Caribbean, a combination of release from top-down control due to loss of the sea urchin *Diadema antillarum* to disease, coupled with overfishing, eutrophication, and destruction of the physical structure of the reef due to hurricanes, led to nearly complete loss of Caribbean corals [3], [11]–[13]. Loss of coral cover due to bleaching or disease can also lead to a coral-algal phase shift [14] since coral death results in available substrate for fast-growing algal species to colonize and eventually dominate the substratum. Loss of herbivorous fish due to overfishing also facilitates this process by allowing algal growth to proceed unchecked, and this can be further exacerbated by addition of nutrients [10].

Benthic coral reef communities are areas of constant competition for space and light [15], [16]. For sessile organisms these battles are a matter of survival and have fundamental consequences for the physical and biological structure of a coral reef community [17]. Despite the abundance of interactions between and among corals and algae, few studies have directly addressed the mechanisms or detailed characteristics of these interactions [15]. It is important to understand how corals and algae compete in order to understand what happens to tip the scales in one direction or another when the ecosystem is perturbed; especially when considering coral-algal phase shifts and possible conservation and restoration strategies.

Corals and algae utilize different physical (sweeper tentacles, messentarial filaments, overtopping, abrasion) and chemical (allelopathy) strategies to compete for and maintain space [16], [18], [19]. Several indirect mechanisms may also exist whereby microbes mediate these competitive interactions. Previous work has shown that dissolved compounds from algae can cause coral death indirectly by enhancing microbial activity [20], [21] and that the addition of dissolved organic carbon (DOC) compounds including those found in algal photosynthates is sufficient to cause coral mortality due to microbial overgrowth [22], [23]. On a reef-wide scale, it has been found that increasing abundance of benthic algae coincides with an increase in the abundance of microbes, including many potential pathogens, which may cause stress to corals and lead to the higher prevalence of coral disease [24]. In addition, algae serve as reservoirs of coral disease [25] and can lead to disease transmission when the two are in direct contact. Despite the various competitive mechanisms that exist there is little information known about the details of these interactions. Does one competitor physically or chemically kill the other and if so how? Are algae able to overgrow live coral? Are microbes involved in these interactions? Experimental evidence has shown that hypoxia can occur on coral surfaces in the presence of algae, suggesting that microbial growth is stimulated [20], however, more data are needed to determine how common hypoxic zones are on the reef.

Hyperspectral imagery is a potentially informative tool for exploring coral-algal interactions. Hyperspectral images are produced by imaging spectrometers, which use an optical dispersing element (e.g. grating or prism) that splits the light into many wavelength bands, which are then detected using 100–1000 s of detectors (e.g. across a CCD chip). In this way, an imaging spectrometer can make spectral measurements of a line. By stepping through one line to the next, a hyperspectral image is built. Hyperspectral imaging is a very active area of research and development, particularly in the area of remote sensing. Airborne hyperspectral imaging combined with Global Information Systems (GIS) is used for agricultural mapping [26], [27], mineral exploration [28], and aerial monitoring of coral reef benthic habitats [29], [30] but has not been used on the scales at which coral reef organisms interact. Multi-spectral imaging has been used to non-invasively monitor diseases in humans [31], [32], suggesting that hyperspectral images may be informative for monitoring tissue changes in corals and algae.

All photosynthetic organisms have absorbance/reflectance spectra that are directly related to their light harvesting pigments. While a significant amount of photophysiology has been conducted on benthic marine algae, almost all photo documentation of corals to date has been done in the visible light range, yet there remains a wealth of information outside of this range that can be captured by hyperspectral imaging. Corals possess characteristic fluorescence profiles [33]–[36] generated by a wide diversity of pigments from the coral animal, their symbiotic dinoflagellates and associated microbes [37], [38]. Despite the prevalence of fluorescent pigments in corals, their role remains an outstanding question. Hypothesized functions include photoprotection [33], [39], [40], enhancement of photosynthesis [41], and quenching of reactive oxygen species [38], [42]. Previous studies show that corals undergoing bleaching have different pigment profiles due to loss of zooxanthellae [43] and changes in coral-associated pigments [44], and that these changes are predictive of coral survival [44]. Hyperspectral imagery can capture changes in host and symbiont pigments at interaction zones and facilitate identification of different organisms at the interaction zone based upon their unique reflectance spectra.

The goals of this study were to determine the prevalence of different coral-coral and coral-algal interactions *in situ*, quantify the oxygen profiles at the boundary layers across interaction zones for a variety of coral and algae, and characterize the hyperspectral signatures of these interaction zones from coral reefs in the uninhabited Southern Line Islands. We identified three main categories of benthic interactions occurring on these healthy reefs: coral vs. coral, coral vs. alga, and alga vs. alga. Upon close examination of different interactions, we identified commonalities and characteristics that were specific to the type of interaction involved. Coral interactions with fleshy algae (turf and macroalgae) were consistently hypoxic, while borders with CCA or other corals were not. Disrupted coral tissue was clearly distinguishable from healthy tissue, algal tissue, cyanobacterial colonization, and bare skeleton. Finally, based on field observations, we found that coral-algal interactions are a constant and widespread feature of healthy coral-dominated reefs.

## Materials and Methods

### Study Site and Specimen Collections

This study was performed during an expedition to the Southern Line Islands, central Pacific in March-April 2009. The islands visited were Vostok (10.1°S, 152.38°W), Starbuck (5.62°S, 155.93°W), Malden (4.017°S, 154.93°W), Flint (11.43°S, 151.82°W), and Millennium (9.94°S, 150.21°W). A variety of corals, algae, and coral-algal and coral-coral interaction zones were collected via SCUBA using a hammer and chisel (see details below). Samples were collected under a Scientific Research Permit issued by the Republic of Kiribati for the period of March 24 – May 5, 2009.

### Underwater Interaction Surveys

Surveys were conducted at 10 m depth on SCUBA to determine the abundance of different types (species, genus or functional groups) of coral-algal interactions and to quantify the outcome of these interactions. A total of five 10 m transects were assessed on the leeward side of Millennium Atoll. Along each of the transect lines a point intercept approach was used whereby every coral that was within contact of the transect line was examined and all algal interactions that occurred on that colony were recorded. The algal species and/or functional group (for turf and CCA) was recorded for every interaction. Additionally divers determined the outcome of the interaction by noting whether the algae was overgrowing the coral, the coral was overgrowing the algae, or if the interaction appeared to be neutral.

### Hyperspectral Imaging

Several coral-algal interaction zones were collected in the field over the duration of the research cruise. All samples were collected with a hammer and chisel, placed in a Ziploc bag, and upon return to the surface were placed in buckets with ambient seawater and returned to the ship. Once shipboard all specimens were kept in 10 liter aquaria with continuous aeration at ambient seawater temperature (∼30°C) in shaded natural light and were imaged within 2–4 hours of return to the ship using the methods described below. The following pairs were imaged: 1) Coral-algae: *Pocillopora verrucosa* vs. *Gracilaria* sp. (n = 4), *Pocillopora verrucosa* vs. *Bryopsis pennata* (n = 6), *Montipora* sp. vs. mixed red turf algae (n = 4), *Pocillopora verrucosa* vs. cyanobacteria (n = 4), and various coral genera (*Favia* sp., *Montipora* sp. or *Pocillopora* sp.) vs. CCA (n = 6); and 2) Coral-coral: *Pocillopora verrucosa* vs. *Montipora* sp. (n = 1) and *Acropora* sp. vs. *Montipora* sp. (n = 1) (Table 1).

**Table 1:** Summary of oxygen microprobing and symptoms from hyperspectral images from coral-algae and coral-coral boundaries in the Southern Line Islands.

	Oxygen at interface (% of seawater background)	Symptoms (moving from algae to coral)
**Coral-algae**		
***Pocillopora verrucosa Gracilaria*** ** sp.**	17–95%; average 68.1% (n = 6)	Algae is proceeded by a cyanobacteria Bleached coral tissue and some bare skeleton at interface Disruption of coral tissue nearest interface (n = 4)
***Pocillopora verrucosa Bryopsis*** ** sp.**	nd	Bare skeleton between algae and coral Distinct white band at interface Coral tissue is peeling off skeleton in patches (n = 6)
***Montipora*** ** spp. Red turf algae**	18–74%; average 44.8% (n = 4)	Algae is proceeded by a cyanobacteria Algal filaments extending over interaction zone Bleached coral tissue (but no bare skeleton) at interface Disruption of coral tissue nearest interface (n = 4)
**Damsel fish territories ** ***Pocillopora verrucosa*** ** Red cyanobacteria**	31–49%; average 40.6% (n = 7)	Algae is proceeded by an unknown green algae At interaction zone, polyps remain intact, calicoblastic tissue is peeling off in patches (n = 4)
**Coral – CCA** ***Favia*** ** sp. ** ***Montipora*** ** sp. ** ***Pocillopora verrucosa*** ** CCA**	85–132%; average 107% (n = 6)	CCA and coral interaction very tight association No coral tissue disruption (n = 6)
**White band disease ** ***Montipora*** ** spp.**	30–47%; average 38.3% (n = 2)	nd
**Coral-Coral**		
***Pocillopora verrucosa Montipora*** ** sp.**	132.8% (n = 1)	Disruption of tissue on *Montipora* spp. (Figure S2; n = 1)
**Coral-Algae-Coral**		
***Acropora*** ** sp. ** ***Montipora*** ** sp.**	nd	Disruption of *Acropora* spp. tissue next to algae (n = 1)

The Resonon PIKA II imaging spectrometer and its associated software, SpectrononPro v.1.15, was used to gather and analyze multibeam images of coral, algae and the interaction zones between them. Light exposure was set using a sheet of white Teflon placed on top of a Petri dish (which eventually held the specimen). After Spectral Focusing and Dark Adjustment the same piece of Teflon was used to record the Response Curve. Once an image was obtained, the mean spectrum of an area of sample was determined using the “Make ROI and Mean” tool in the accompanying SpectrononPro software. In order to compare spectra between samples, several normalization techniques were tested to eliminate small differences in the absolute relative reflectance of similar spectra. The simplest and most straightforward was to calculate the slope between every pair of wavelengths across the spectrum and plot (i.e. the first derivative of the reflectance). A number of image processing algorithms contained in the SpectrononPro software were tested for applicability to the corals and algae that were imaged. Many of these tools were developed for agricultural use, so were tested for usefulness when comparing marine photosynthetic organisms. The algorithms were: Green-orange-chlorophyll (GOC), Color Infrared (Color IR), Simple Ratio (SR) [45], Normalized Difference Vegetative Index (NDVI) [46] and Atmospherically Resistant Vegetative Index (ARVI) [47], in addition to the default True Color rendering. True Color, GOC, and Color IR yield false-color images. True color uses the three bands red (640 nm), green (550 nm), and blue (460 nm), GOC uses the three bands green (515 nm), orange (575 nm), and chlorophyll (685 nm), and Color IR uses the three bands green (550 nm), red (650 nm), and infrared (IR; 860 nm) with a 2% Stretch Contrast Enhancement. SR, NDVI, and ARVI are black-and-white mono (1-layer) images from a data cube with a 2% Stretch Contrast Enhancement (i.e. set the darkest 2% of pixels to black, the brightest 2% of pixels to white, and stretch the remaining 96% of values between black and white). In order to determine pixel values, SR uses the ratio between IR (800 nm) and red (680 nm), NDVI uses IR (800 nm) and red (680 nm) as input for the formula (IR - red)/(IR + red), and ARVI uses the bands IR (800 nm), red (680 nm), and blue (450 nm) as input for the formula (IR−2*red + blue)/(IR+ 2*red − blue).

### Dissolved Oxygen Measurements

To determine how dissolved oxygen concentration changed across several different types of coral-algal interaction zones, dissolved oxygen (DO) levels at the boundary layer were measured using an oxygen microprobe (Unisense; Aarhus C, Denmark). The microprobe was calibrated before each interaction zone was measured using aerated seawater to obtain the atmospheric saturation level of dissolved oxygen (100% DO), followed by a solution of 0.1 M sodium hydroxide and 0.1 M sodium ascorbate for the 0% DO reference point. Once calibrated, the probe was lowered to the boundary layer above the surface of the algae, coral, or interface zone under a Leica MZFLIII dissecting microscope. Five random points from within each zone were measured for a total of at least 10 seconds per point, and a measurement of aerated seawater was taken between each point. Data were recorded using the Unisense SensorTrace Basic v.1.13 software.

### Dissolved Oxygen Data Analysis

Ten recordings at each point were averaged to obtain the minimum or maximum level of dissolved oxygen (DO) for each point probed. The percent DO was calculated relative to the measurement of aerated seawater (100% DO) prior to each measurement of the sample point. Normalization was carried out to account for drift of the instrument signal over the course of the measurements. The average of the percent DO from the 5 points taken within a zone was calculated to get the average maximum or minimum percent DO for the given sample zone (i.e. coral, algae, or interface). Several different coral-algal species (or functional groups for turf algae and CCA) interactions were examined including: 1) Coral-algae: *Pocillopora verrucosa* vs. *Gracilaria* sp. (n = 6), *Montipora* sp. vs. red turf algae (n = 4), *Pocillopora verrucosa* vs. cyanobacteria (n = 7); various coral genera (*Favia* sp., *Montipora* sp. or *Pocillopora* sp.) vs. CCA (n = 6), and *Montipora* sp. vs. white band disease (n = 2), and 2) Coral-coral: *Pocillopora verrucosa* vs. *Montipora* sp. (n = 1) (Table 1). As with the hyperspectral images, dissolved oxygen measurements of interfaces were taken within 2–4 hours of removal of samples from the reef. The non-parametric Man-Whitney test was used to compare DO levels because of uneven sample sizes. Significance was assessed by an asymptotic 2-tailed test with p<0.05.

## Results and Discussion

**Figure 1:**
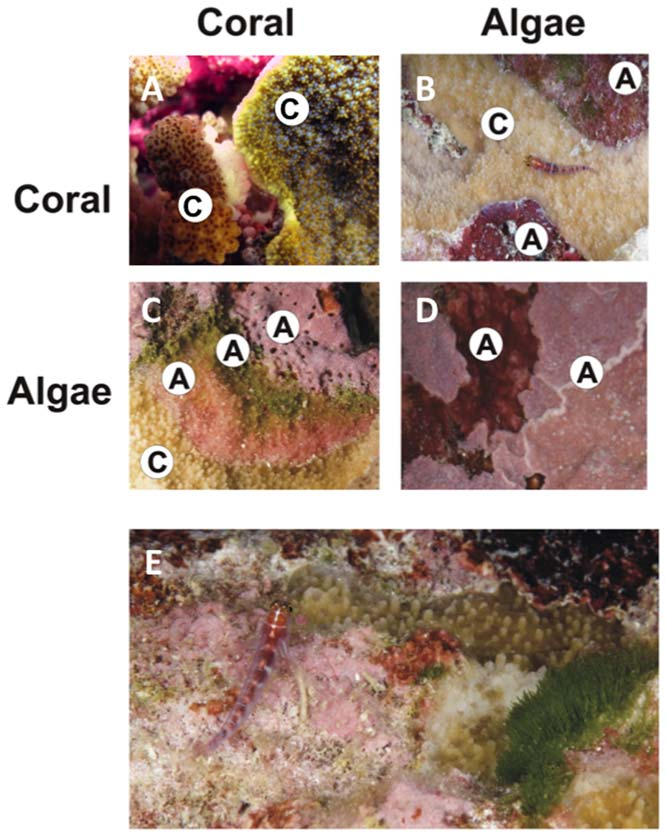
Examples of boundaries between coral and algae. A) *Pocillopora* sp. vs. *Montipora* sp., B) algae vs. *Montipora* sp., C) *Montipora* sp. and various algae, D) crustose coralline algae vs. crustose coralline algae, and E) diverse interactions including coral, fleshy algae, crustose coralline algae, and other invertebrates.

We identified a wide variety of interactions between benthic organisms on the reefs of the Southern Line Islands. Overall, coral vs. coral (Figure 1A), coral vs. algae (Figure 1B, C), and algae vs. algae (Figure 1C, D) were the most common. This island archipelago has relatively low biodiversity with some 50 species of coral, 10 common species of macroalgae, many species of turf algae, and at least 5 species of crustose coralline algae (CCA). The low level of diversity still provides for an enormous number of possible different paired interactions between groups of benthic organisms, which is further complicated by the fact that most interaction zones include multiple organisms. For example, in Figure 1C the algal interface actually consists of at least 3 algal types (e.g. a green alga, a red alga, and a CCA) and Figure 1E shows how extremely complex these zones can be with multiple species of algae and corals intermingled.

**Figure 2:**
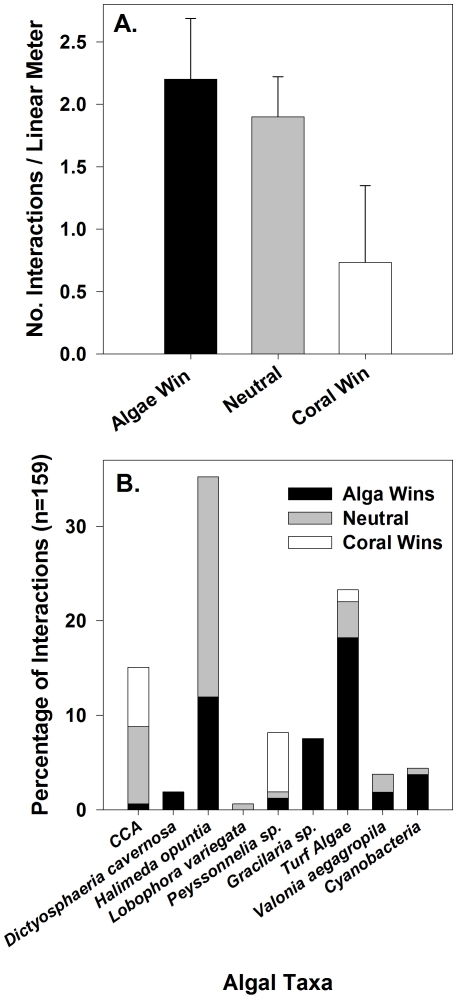
Summary of interactions between corals and algae from surveys of Millennium Atoll. A) Total number of interactions between corals and algae, B) outcome of coral-algal interactions by algal group.

Benthic transects to quantify coral-algal interactions found on average 4.57 interactions per linear meter (Figure 2A). Of these, over half of the interactions were either neutral or were designated as coral-dominated (Figure 2A), while in other cases different genera/functional groups of algae appeared to out-compete the coral. For example, the fleshy red macroalga *Gracilaria* sp. was observed at every stage of overgrowth of *Pocillopora* colonies, from initial algal growth up from the base of the coral head over live coral tissue to dead colonies completely overtaken by the alga. The remoteness of the study site prohibited time series examinations of these interactions, yet the presence of *Gracilaria* sp. at various stages of overgrowth of living coral colonies was a clear indication of competitive dominance of this alga. Furthermore, close up observations revealed several stages of algal advance, including fronds directly in contact with live coral tissue and fronds surrounded by areas where the coral tissue had died. In contrast, other types of algae such as CCA or *Peyssonnelia* sp. were found being overgrown by corals in many cases (Figure 2B). These observations demonstrate the variability between different coral-algal species/functional group interactions.

### Spectral Analysis of Corals and Algae

The spectral signatures of corals and algae were contrasted to determine the consistency of the spectra between individuals from the same group, and whether hyperspectral images were therefore useful for characterization of interaction zones. While hyperspectral imaging has been used in other studies to ascertain organisms over large spatial scales, it has never been used to discriminate among organisms on the scale examined here. Furthermore, this is the first study to use hyperspectral imaging as a tool for characterizing competitive interactions between different photosynthetic reef organisms (Table 1). As a test case, several crustose coralline algal (CCA) specimens were imaged and the mean spectrum across each was calculated. The CCA spectra were very similar to each other (Figure S1A). A number of normalization techniques were tested to eliminate differences in absolute reflectance values. The simplest and most straightforward was to calculate the slope between every pair of wavelengths across the spectrum and plot (i.e. the first derivative; Figure S1B). This transformation normalized the data and highlighted the most important components (i.e. differences) of the spectra.

**Figure 3:**
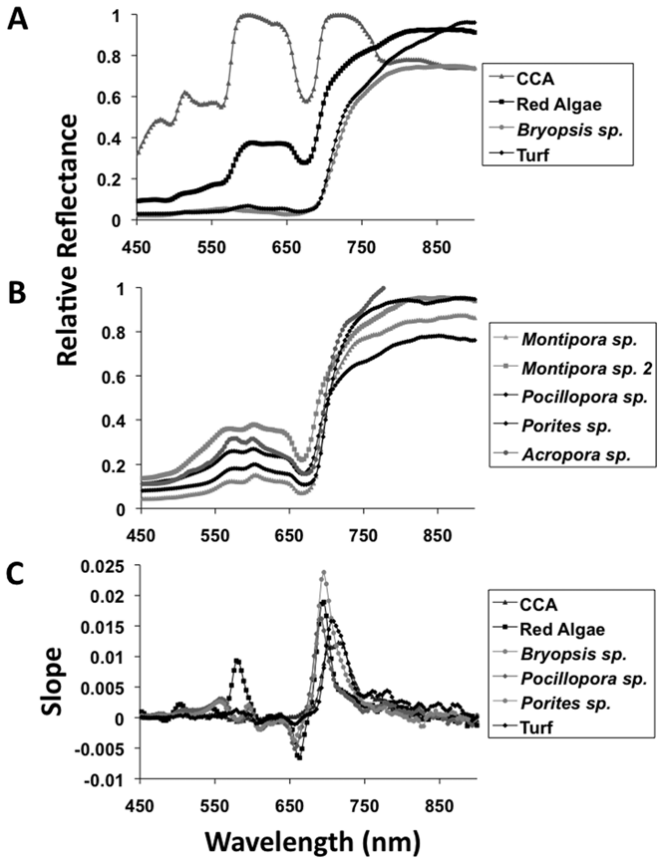
Comparison of spectra from different algal and coral groups. A) Relative reflectance of different algae: CCA, red alga, *Bryopsis* sp., and turf algae. B) Relative reflectance of 4 different corals: *Montipora* sp. (2), *Pocillopora* sp., *Porites* sp., and *Acropora* sp. C) Slope of coral and algal reflectance spectra, including CCA, red alga, *Bryopsis* sp., *Pocillopora* sp., *Porites* sp., and turf algae.

The main groups of reef algae encountered (e.g. encrusting CCA, fleshy reds, fleshy greens (*Bryopsis* sp.), and turf algae) were imaged and compared to ground truth this novel method (Figure 3A). Each group had a characteristic spectrum, clearly distinguishable using the hyperspectral data. This result was expected given that different types of algae contain a variety of pigments, which would lead to clear differences in reflectance spectra among taxa. Different coral genera were then imaged with the hyperspectrometer. The expected maximum reflectance wavelengths for corals were 575 and 685 nm [35], [36], and were readily seen in the coral spectra generated by the hyperspectrometer (Figure 3B). The reflectance curves collected were similar to the spectra described in previous studies for various coral species around the world [29]. When compared, the reflectance spectra from different coral genera were similar and were not clearly distinguishable from each other (Figure 3B), unlike the different algal groups (Figure 3A).

To test if spectra varied between corals and algae, two selected coral spectra (*Pocillopora* sp. and *Porites* sp.) were compared to the different reef algae. As shown in Figure 3C, it is easy to distinguish between these major groups at the fine scale using the spectra alone. While corals are spectrally very similar to each other due to their symbiotic dinoflagellate and host pigments, they are distinct from the major algal groups. Together these results show that hyperspectral signatures are sufficient to broadly differentiate between different types of algae and corals. Furthermore, we can now compare spectra from healthy corals and algae to determine if the spectra (e.g. pigment presence or patterning) changes or breaks down when the two organisms are in direct contact. Changes in pigmentation or tissue structure revealed by hyperspectral images along interaction zones may be a symptom of stress due to competition between the two groups.

### Corals Versus Algae

**Figure 4:**
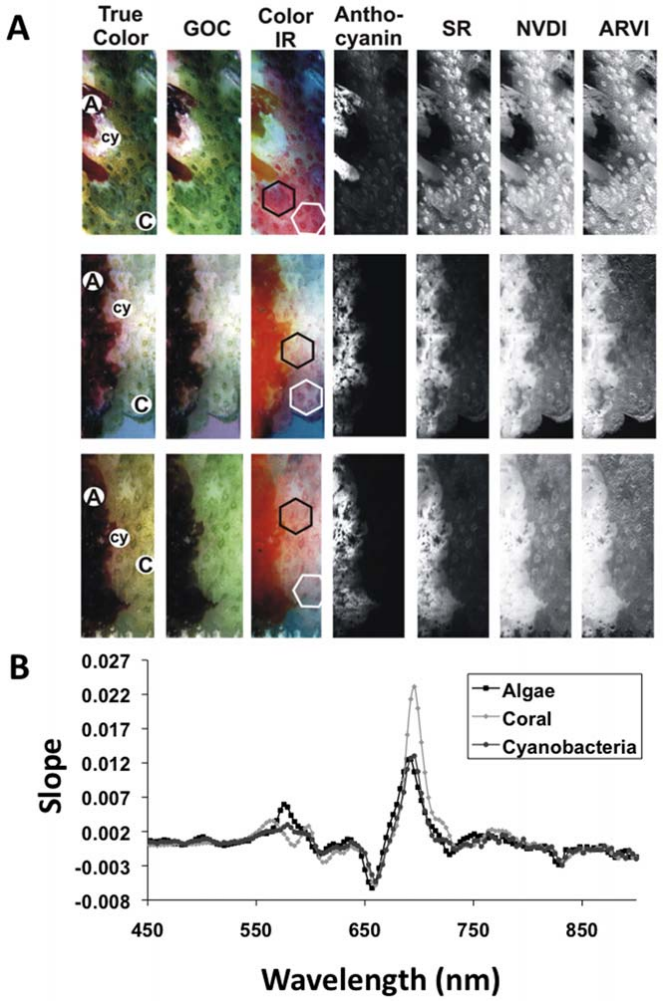
Different renderings of hyperspectral images of the interaction zones between the coral *Pocillopora* sp.and red alga *Gracilaria* sp. A) The location of algae (**A**), coral (**C**) and cyanobacteria (**cy**) are indicated in the True Color image. The color coding is determined for **True Color** using the three bands red (640 nm), green (550 nm), and blue (460 nm); **GOC** using the three bands green (515 nm), orange (575 nm), and chlorophyll (685 nm); **Color IR** using the three bands green (550 nm), red (650 nm), and infrared (IR; 860 nm); **SR** (Simple Ratio) using the ration between 800 nm and 680 nm; **NVDI** (Normalized Vegetative Density Index) using IR (800 nm) and red (680 nm) in the formula (IR − red)/(IR + red); and **ARVI** (Atmospherically Resistant Vegetative Index) using the bands IR (800 nm), red (680 nm), and blue (450 nm) entered in the formula (IR − 2*red + blue)/(IR+ 2*red − blue). Each rendering uses a 2% Stretch Contrast Enhancement. B) The slope of the average relative reflectance for the algae, coral, and cyanobacteria imaged in A. Areas used to determine the average reflectance are indicated by hexagons in the IR rendering.

**Figure 5:**
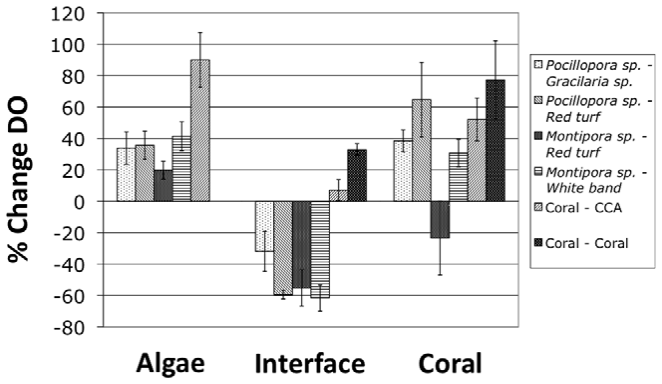
Dissolved oxygen profiles of coral interaction zones. Interactions measured were *Pocillopora verrucosa* vs. *Gracilaria* sp. (n = 6), *Pocillopora verrucosa* vs. red turf algae (n = 7), *Montipora* spp. vs. red turf algae (n = 4), *Montipora* sp. vs. white band (n = 2), coral vs. CCA (n = 6), and coral vs. coral (n = 1).

Interactions between corals and algae were highly abundant across the coral reefs surveyed (Figure 2). A series of these interactions involving different species were imaged with the hyperspectral camera and dissolved oxygen levels were measured across the surfaces of the interaction zones (Table 1). Comparisons of hyperspectral images of coral-algal borders indicated that interaction zones shared common characteristics. Interactions with fleshy algae (e.g. *Gracilaria* sp., *Bryopsis* sp., and various turf algae) were typically characterized by bleached or disrupted coral tissue near (mm scale) the interface (Table 1). These areas were often pale, indicating loss of zooxanthellae from live coral tissue, and in many cases the characteristic patterning of coral pigments and polyps was altered and the tissue appeared damaged. In areas where skeleton was revealed following coral tissue death, cyanobacteria were observed (Figure 4A, Table 1). We found that at the point of contact for all of the interactions between corals and fleshy turf algae or macroalgae there was a zone of hypoxia, but the degree of hypoxia varied depending on the type of alga involved (Figure 5). A Mann-Whitney test recognized four groups in the data. For example, coral interaction zones with *Gracilaria* sp. were less hypoxic (p<0.05) on average than corals interaction zones with turf algae (Figure 5). The cause of the observed hypoxia remains to be determined, and may be due to respiration of the coral tissue itself, microbial respiration on the surface of the coral, or a combination of the two. The degree of coral mortality likely affects the level of hypoxia, and the degree of coral tissue mortality appears to be related to the functional group and most likely the species of algae with which it comes in contact (Table 1).

In contrast, the interactions between corals and crustose coralline algae (CCA) did not show any evidence of hypoxia (Figure 5). Hyperspectral images of coral-CCA interactions showed that corals and CCA were in close association, yet there were no areas of cleared coral skeleton and coral tissue was not disrupted or visibly stressed near the interface (Table 1). This may be because CCA do not stimulate or alter microbial communities associated with the coral tissue, or do not directly damage or kill the coral tissue through release of allelochemicals. These data indicate that there are at least two distinct mechanisms of interaction between corals and algae. In the case of at least some fleshy turf and macroalgae, coral tissue structure and pigmentation were clearly disrupted and dominated by respiration, indicating microbial overgrowth and clear stress to the coral animal, while corals interacting with CCA showed no signs of stress.

As a test case, interaction zones between the coral *Pocillopora* sp. and a red alga *Gracilaria* sp. were characterized in detail using hyperspectral imaging. These images are data rich and there is a vast literature of different techniques for processing hyperspectral images. Here we used renders and utilities built into the supporting software Spectronon Pro to identify a process that best displayed the differences in the interaction zones. Figure 4A shows some of the more visually informative processing. In general, the advancing *Gracilaria* sp. branches were preceded by a thin line of cyanobacteria, followed by an area of bare skeleton and then disrupted coral tissue (Figure 4A). The oxygen levels at the interface were somewhat variable. Regions of apparently bare skeleton following coral tissue death had oxygen levels typically near or just below ambient (data not shown), while areas of disrupted coral tissue were hypoxic (Figure 5).

Because both components of coral-algal interactions are photosynthetic, a number of image processing algorithms normally used for agriculture were also tested. Of these, specific pigments such as anthocyanin (similar reflectance peaks as the red algal phycobilin pigments, Figure 4A) and carotenoids (not shown) were useful for distinguishing changes along the interaction zone and between the coral and the algae. The GOC and Color IR renderings helped visualize the different components of the interaction zone (Figure 4A). Colonization of exposed skeleton at the interface by cyanobacteria was seen by hyperspectral imagery, and the reflectance spectra for cyanobacteria were clearly distinguished from the spectra of the *Gracilaria* sp. and *Pocillopora* sp. (Figure 4B). Renderings of Simple Ratio (SR), Normalized Vegetative Density Index (NVDI), and Atmospherically Resistant Vegetative Index (ARVI) all showed similar patterns (Figure 4A). The algal component had the highest values (white coloring), while the coral showed patterning likely due to the distribution of zooxanthellae. Except for anthocyanin, all renderings clearly showed that advancing algal fronds in direct contact with coral tissue cause a clear disruption of the natural patterns of the coral polyps and pigmentation in the area surrounding the point of contact (Figure 4A, top row).

### Corals Versus Corals

Two types of coral-coral interaction zones were found. In an active border region, one coral was disrupting the tissue of the other (**dt** = disrupted tissue, Figure S2). The spectra of these boundaries showed no evidence of algal colonization, and these borders were not hypoxic (Figure 5). Competition between corals is known to involve mesenterial filaments, sweeper tentacles and nematocysts [48], [49], and so would be expected show a distinct oxygen profile from coral-algal competition. One note of interest, in the *Pocillopora* sp., individual polyps appear to be releasing large quantities of mucus (labeled as **m** in Figure S2A). Constant activation of a stress response by competition is likely affecting the overall health of the coral, and previous work has shown that competition between corals can reduce growth and fitness [50].

The second type of coral-coral interaction zone can actually be defined as a coral-algae-coral zone. In this case algae have colonized the area between the two corals. On Millennium Atoll, the majority of apparent coral-coral interactions were actually found to be coral-algae-coral interactions upon close examination (Table 1, Figure S2B). It appears that as corals compete with one another, an area between the two competitors is cleared of live tissue, which is then colonized by opportunistic algae and likely microbes. Previous work has found that competing corals constantly advance and retreat, leaving areas of cleared space in between the two colonies as they recover from competitive interactions [51]. It is unknown if the presence of algae between the two colonies is detrimental, beneficial, or neutral for the competing corals.

### Conclusions

Interactions between corals and algae were a widespread feature of the near-pristine coral reefs of the Southern Line Islands. Hyperspectral imagery and oxygen profiles of coral-algal interaction zones demonstrated that these interactions have characteristic profiles that depend on the species and functional group of algae involved. Coral interaction zones with fleshy algae (e.g. red and green macroalgae and turf algae) were characterized by disrupted coral tissue near the interface, and were consistently hypoxic. Hypoxia suggests that respiration by microbial activity may dominate these areas. For example, human wounds are often hypoxic as a result of microbial respiration, which hinders the host immune response and slows or prevents wound healing [52]. While we cannot rule out coral respiration as a cause of hypoxia, in experimental manipulations, algae placed near corals led to hypoxia on the coral surface and mortality, which was eliminated by the addition of antibiotics, indicating that coral death was microbially mediated [20]. In contrast to fleshy algae, we found that coral interactions zones with CCA were not hypoxic, and coral tissue at the interface appeared normal. These findings indicate that competitive interactions between reef building corals and fleshy algae or CCA likely have fundamentally different consequences for corals and reef communities as a whole. CCA have been found to be beneficial for corals in many cases by providing a settlement substrate and metamorphosis cue for coral larvae [53], [54] and by helping maintain the structural stability of reefs [55]. On the other hand, fleshy algae may be a constant source of stress for corals. On reefs where algae are released from grazing pressure and/or nutrient limitation, fleshy algae dominate [3], [9]–[11], and their ability to disrupt live coral tissue, as observed in this study, likely plays an important role. More data are needed to determine how these patterns vary between different fleshy and calcified algal taxa.

The complexity of coral interactions was further revealed upon close examination with hyperspectral imagery. For example, the majority of apparent coral-coral interactions were actually coral-algae-coral interaction zones. In addition, the red alga *Gracilaria* sp. appears to rapidly advance over some species of coral, directly disrupting the tissue and clearing areas of skeleton that are subsequently colonized by cyanobacteria. These observations demonstrate that some algae (e.g. cyanobacteria) will opportunistically colonize available space, while others actively overgrow corals (e.g. *Gracilaria* sp.). Interactions with turf algae were not as dramatic and did not show areas of cleared coral skeleton, but turfs were still disruptive to adjacent coral tissue although the advance appeared much more gradual and variable than that of *Gracilaria* sp. The advance of turf algae was likely limited by intense grazing pressure on these reefs as turf algae are readily consumed by reef herbivores. Although grazing rates of herbivores were not measured, the high abundance of herbivorous fish and general low abundance of algae on Millennium Atoll suggests that grazing pressure may be limiting algal growth (data not shown).

Hyperspectral imagery was confirmed as a useful tool to visualize the small scale interaction zones between corals and algae, and extends the spectral range encompassed in analysis from previous studies of coral spectra [34]–[36], [29], [56]. This technique clearly identified the players involved in various coral and algae interactions, and revealed changes in tissue patterning and pigmentation at the interaction zone. Hyperspectral imagery is currently being developed for remote monitoring of coral reef benthic communities, but could be expanded as a useful tool for future monitoring of coral reefs by rapidly characterizing the abundance of coral-algal competition borders *in situ* at the fine scale. Hyperspectral imaging technology is not currently available for underwater fine-scale analysis, but multi-band technology encompassing important wavelengths indicative of corals, algae and microbes (e.g. cyanobacteria) is a viable next step.

This is the first study to describe the physiological characteristics of different types of coral-algal interactions on a coral reef. The combination of hyperspectral imagery with dissolved oxygen measurements of these interactions indicate that coral-algal interfaces vary among species and in overall characteristics. Interaction zones between corals and at least some fleshy algae appear to be detrimental to corals. On the other hand, CCA do not appear to disrupt corals in the same manner and in fact facilitate the maintenance of coral reefs by providing settlement substrate for corals and solidifying the reef structure. The results of our study show that some fleshy algae, a highly diverse group of benthic primary producers that includes macroalgae as well as turf algae, can have competitive advantages over slower growing reef building corals. These types of algae can be disruptive to live coral tissue, and are increasingly abundant on impacted coral reefs worldwide [1], [3], [9]–[11], [14], [57]. Understanding the drivers that shift competitive dominance towards fleshy algae remains an outstanding research question, the answers to which are important for developing effective management, conservation and restoration strategies for coral reefs.

## Supporting Information

Figure S1 Average reflectance spectra from crustose coralline algae (CCA). A) Relative reflectance of 5 different CCA specimens. The CCA fragments were overexposed as evidenced by peaks that are cut off at 1 (630 nm; 730 nm). B) Slope (first derivative of reflectance spectrum) of the 5 CCA specimens in A.
(2.43 MB TIF)

Figure S2 Two types of interaction zones between corals. A) Active coral interaction zone where one coral is attacking another and damaging the tissue with mesenterial filaments. B) Interaction zone between two corals where algae has established itself between the two competing corals.
(8.90 MB TIF)
