# Supplementary figures and images for: Hyperspectral and Physiological Analyses of Coral-Algal Interactions
Source: PLoS One. 2009 Nov 26;4(11):e8043. doi: 10.1371/journal.pone.0008043 (PMC2778555; doi:10.1371/journal.pone.0008043)

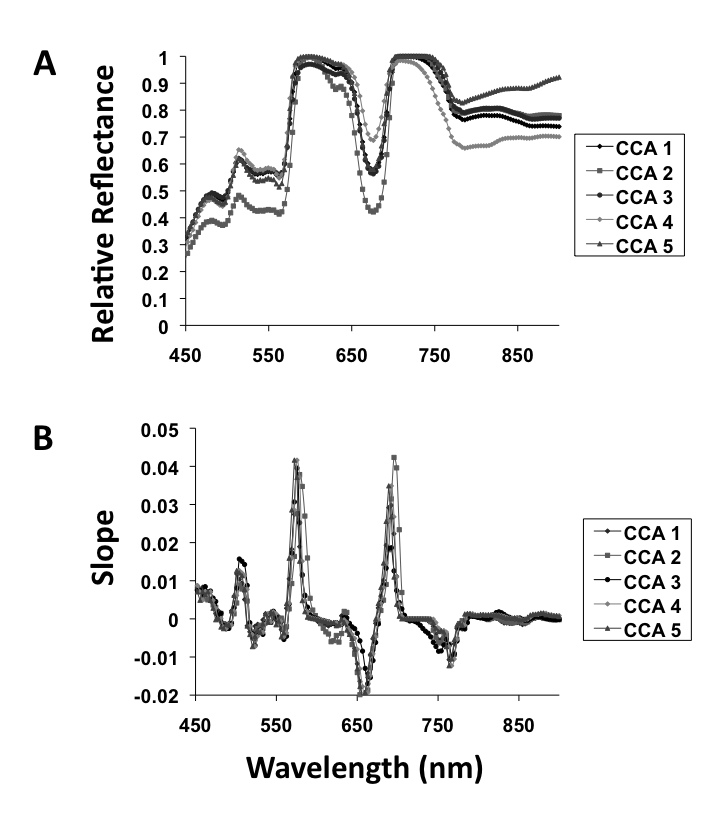

Supplement: Figure S1 — Average reflectance spectra from crustose coralline algae (CCA). A) Relative reflectance of 5 different CCA specimens. The CCA fragments were overexposed as evidenced by peaks that are cut off at 1 (630 nm; 730 nm). B) Slope (first derivative of reflectance spectrum) of the 5 CCA specimens in A. (2.43 MB TIF) [file pone.0008043.s001.tif]

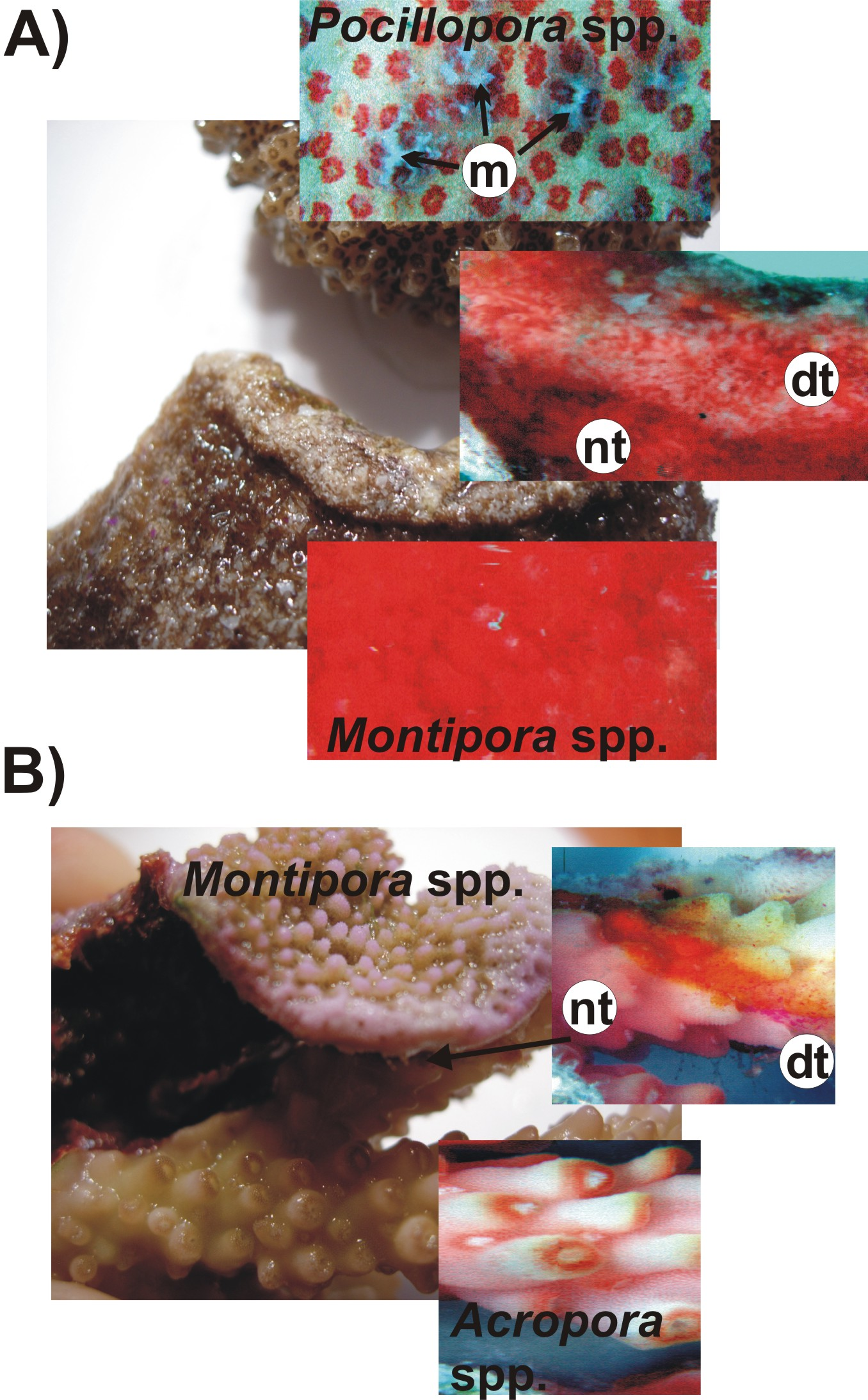

Supplement: Figure S2 — Two types of interaction zones between corals. A) Active coral interaction zone where one coral is attacking another and damaging the tissue with mesenterial filaments. B) Interaction zone between two corals where algae has established itself between the two competing corals. (8.90 MB TIF) [file pone.0008043.s002.tif]
